# Molecular paleontology as an exciting, challenging and controversial field

**DOI:** 10.1093/nsr/nwaa001

**Published:** 2020-01-07

**Authors:** Yanhong Pan

**Affiliations:** State Key Laboratory of Palaeobiology and Stratigraphy, Nanjing Institute of Geology and Palaeontology and Center for Excellence in Life and Paleoenvironment, Chinese Academy of Sciences, China

Molecular paleontology is the study of ancient complex biomolecules associated within deep-time fossils, which may provide important information for understanding the organisms’ evolution and fossilization process at the molecular level, as well as facilitating the recognition of preserved biomarkers in order to identify life on other planets [1,2]. In the paper by Bailleul *et al.* [3], the authors report the discovery of well-preserved subcellular structures morphologically consistent with nuclei and chromosomes in histological ground sections from skull bones of an Upper Cretaceous baby dinosaur. However, as the authors have stated that morphology alone is insufficient to diagnose any cellular or subcellular structures, they conducted further histochemical (Alcian blue stain for extracellular matrix of cartilage; PI and DAPI stains for chemical markers consistent with DNA) and immunological (antibodies against avian collagen II) tests. All tests were positive, providing strong evidence suggesting *in situ* preservation of extracellular matrix components, and even endogenous nuclear materials. This case study reveals interesting evidence of exceptional preservation in fossils at both molecular and morphological levels, and opens the door for extensive further research, making the argument that DNA sequencing is worth exploring. The present study is significant for at least two reasons. First, it demonstrates that fossils displaying well-preserved cellular and subcellular structures may also preserve molecular information. Second, this study clearly shows that the search for ancient biomolecules should not be constrained by the so-called temporal limit, e.g. the assumption that proteins cannot survive in the fossil record beyond ∼1 Ma and ∼100 000 years for DNA. The preservation mechanism of soft tissues in fossils is far more complex than we have observed in modern environments through taphonomy experiments.

The last decade has borne witness to numerous discoveries that have provided mounting evidence of molecular preservation in deep-time fossils, e.g. proteins, certain carbohydrates such as chitin and cellulose, sterol lipids and pigments [4–8]. As this is a relatively new field, searching for ancient molecules in fossils is full of challenges, e.g. inevitable contaminations, immature techniques and unknown modifications of molecules over geological time. Thus, new discoveries in molecular paleontology are often accompanied by controversy. However, controversy is a crucial part of scientific progress and incorrect ideas do less harm to science than false evidence. To promote the discipline of molecular paleontology, we need to overcome bias and prejudice from colleagues in and outside the field, particularly with regard to the presumed ‘preservational limit’. Another common critic plaguing discoveries of ancient biomolecules concerns the repeatability of the results; however, it is an obvious bias to declare that the result cannot be replicated if different techniques or methods are used.

Finally, it is recognized that ‘exceptional claims require exceptional evidence’ and multiple independent lines of evidence should be provided in support of any new discovery of ancient biomolecules in deep-time fossils as done by Bailleul *et al.* [3]. With the recent and rapid technological developments, we are optimistic that the field of molecular paleontology will grow rapidly and provide additional evidence of unexpected fossil biomolecules from geological ages previously held to be too old for such biomolecules to survive.
